# Application of Municipal Biowaste-Derived Products in Tomato Cultivation for Enhanced Fruit Quality Attributes and Nutritional Profile

**DOI:** 10.3390/plants14203212

**Published:** 2025-10-19

**Authors:** Giannis Neofytou, Antonios Chrysargyris, Marianna Christodoulou, Enzo Montoneri, Michalis Koutinas, Nikolaos Tzortzakis

**Affiliations:** 1 Department of Agricultural Sciences, Biotechnology and Food Science, Cyprus University of Technology, Limassol 3603, Cyprusa.chrysargyris@cut.ac.cy (A.C.); 2 Department of Chemical Engineering, Cyprus University of Technology, Limassol 3036, Cyprusmichail.koutinas@cut.ac.cy (M.K.); 3 Department of Agricultural, Forest and Food Sciences, University of Turin, 10124 Torino, Italy

**Keywords:** bioproduct, municipal biowaste, tomato, plant growth, fruit quality

## Abstract

Enhancing plant nutrient use efficiency, yield, and quality without compromising sustainability remains a critical challenge in agriculture. Utilization of materials such as biowaste derivatives as alternatives to conventional agrochemicals (e.g., fertilizers, biostimulants) can be leveraged to optimize crop productivity and resilience while adhering to sustainable practices. A soluble bioproduct (BP), isolated from the hydrolysis of anaerobic digestates derived from organic residues of urban waste, was examined for its capacity to enhance tomato (*Solanum lycopersicum*) production and quality. Five basal fertilization treatments were applied: conventional (CF), conventional/organic (CF + OF), bioproduct at 150 kg ha^−1^ (BP), and conventional/BP at 150 and 300 kg ha^−1^ (CF + BP, CF + 2BP), without or with supplementary fertigation (SF). The experiment was arranged in a Randomized Complete Block Design. Intermediate plant growth under BP was comparable to CF, while their combination enhanced growth parameters. However, addition of BP to CF did not affect final plant growth, biomass, and yield compared to CF alone, though non-significant reductions of height (5.37%), leaf number (15.89%) and fresh weight (36.09%) were observed with BP alone. The same treatment reduced intermediate leaf macronutrients (N, K, Na), whereas this was ameliorated with CF + BP. The use of BP without fertigation enhanced final P content in leaves and roots. However, fruit P declined, reflecting delayed P availability and translocation. The use of BP induced plant stress responses, accompanied by stimulation of phenolic and antioxidant accumulation in leaves, with fruit exhibiting comparable increases only without fertigation. Fruit lycopene and total soluble solids were enhanced by CF + BP, with fertigation mediating differences. Combined CF and BP application promoted tomato fruit quality, without diminishing growth, while the performance of BP alone was improved with supplementary fertigation to maintain tomato growth, yield, and quality.

## 1. Introduction

Modern lifestyles, rapid urbanization, and a growing population have resulted in increased municipal solid wastes (MSW) production, which represents a pressing environmental issue [1,2]. In fact, MSW generation is projected to reach approximately 2.2 billion tonnes per year by 2025 [3]. The large amount of waste is also associated with higher disposal costs [4]. About 18% to 60% of MSW comprises biowaste, which includes food and gardening wastes. These residues frequently end up in landfills without any proper treatment, posing significant impacts on the environment and public health due to methane and bacteria build up [4,5]. In addition, leakage of leachate produced by MSW can pollute soils and groundwater, potentially impacting human health through the food chain [6]. This highlights the need for proper management practices to reduce environmental impacts and to recover significant resources within the concept of a circular economy.

Agricultural production has expanded drastically to accommodate the increasing food demands of the global population. This was enabled in part by the intensification of agricultural inputs such as agrochemicals and fertilizers. However, this intensive use of synthetic fertilizers has prompted serious environmental and geopolitical issues [7,8]. Fertilization is a major driver of climate change, contributing up to 20% to the total greenhouse gas emissions budget [9]. Emissions originate directly from fertilizer application (e.g., emission of gaseous nitrogen compounds) and indirectly from the large energy inputs required for their production [10]. In addition, the extensive use of synthetic fertilizers causes nutritional imbalances and diminished soil fertility, causing a reduction of soil organic matter and soil organic carbon [9]. At the same time, fluctuations in the costs of fertilizers increase the cost of food production, posing risks to both food security and safety [11]. To address these issues, circular economy approaches have been proposed to reduce the dependence on synthetic chemical fertilizers while closing nutrient loops, mitigating pollution, and ensuring climate-neutral and sustainable growth [12,13]. This involves the consideration of materials and management practices that enhance crop production, improve soil fertility and ultimately reduce the impact of agricultural production on the climate [9].

The concept of a circular economy emphasizes the transformation of production chains into closed-loop processes that reduce waste and increase resource efficiency [14]. In this context, the valorization of waste could be useful in creating value-added products for use in agriculture [9]. In particular, municipal biowaste represents a sustainable feedstock due to its abundance within urban settings and its negative cost, as collection costs are already covered by tax payers [5,15]. Additionally, waste valorization also decreases the amount of landfilled waste and direct or indirect pressures on ecosystems [16]. The valorization of biowaste consists of anaerobic and aerobic fermentation processes for the production of digestates and compost [13]. Particularly, biowaste can be processed through chemical hydrolysis to obtain bioproducts (BPs) that are employable in agriculture as organic fertilizers, biostimulants, biopolymers, and antifungal compounds [5,15,17]. These BPs are derived from the hydrolysis of various feedstocks, including digestates of anaerobic fermentation of food waste, composted gardening residues, sewage sludge, and crop residues [18]. Interestingly, BPs contain a lower carbon/nitrogen (C/N) ratio compared to compost, and thus can be applied at lower rates as soil amendments [19].

Recently, research has demonstrated the efficacy of BPs as biofertilizers and biostimulants. Several studies utilized BPs at diverse rates ranging from 50 to 500 kg ha^−1^ for the cultivation of various vegetable crops including tomatoes (*Solanum lycopersicum* L.), peppers (*Capsicum annuum* L.) common beans (*Phaseolus vulgaris* L.) [20,21,22], and ornamentals including hibiscus (*Hibiscus moscheutos* L. subsp. *palustris*) and lantana (*Lantana camara* L.) [5,23]. Relative to the plant species, these studies observed increases in plant performance indicators by as much as three orders of magnitude compared to their respective controls [18]. The positive results of BPs used in plant growth can be associated with influences on plant uptake and the availability of nutrients, enhancement of water use efficiency (WUE) and nitrogen use efficiency (NUE), a reduction of nitrate lixiviation, and enhancement of physiological activities [5,24]. However, results may vary depending on the specific species, growth conditions, and the high variability in the composition of the materials used. Additionally, without a well-defined understanding of the action mechanism for the observed performance of BPs, its utilization remains challenging. Nevertheless, the substantial nutrient input of mineral fertilizers under these studies may render the specific effects of the BPs applied less apparent [18].

The utilization of BPs as organic fertilizers and biostimulants has shown great potential in supporting crop growth and productivity [13]. Prior results suggest that the use of BP can significantly influence plant growth and yield, either applied alone or as a supplement to conventional fertilizers. The present work was commissioned to assess the effect of BPs produced using compost of digestate from anaerobic fermentation of food waste and of gardening and trimming residues (CVD) on the growth and yield of tomato (*S. lycopersicum* L.) cultivated in soil under greenhouse conditions. The scope of this research was to investigate the effects of BP as a supplement of conventional fertilizers and possible replacement of organic fertilization used in crops, examining the crop’s performance and soil characteristics. Additionally, BP alone application was used as a means of discerning BP’s independent effects, isolating its contribution from that of conventional fertilizer inputs. The BP was used at different rates when combined with conventional fertilization or used as a sole amendment; the control treatment was conventional fertilization alone. To properly assess the long-term efficacy of the basal fertilization regimes, basal fertilization applications were divided into treatments with or without supplementary fertigation throughout the cultivation period. This design enables the identification of the agronomic efficiency of BP, while the thorough evaluation of parameters related to plant growth, productivity, antioxidant activity, stress responses, nutritional profiles, and quality traits allows the elucidation of its influence on plant physiology and fruit quality.

## 2. Results

### 2.1. Soil Physicochemical Parameters and Leachates Characterization

Selected soil properties of the various fertilization treatments following the end of the cultivation period of tomato plants are presented in Table 1. Under no supplementary fertigation, BP treatment significantly increased soil pH to 7.97, which is 3.4% higher than the lowest pH (7.71) under CF + BP. However, the same treatment reported the lowest soil EC (2.32 mS cm^−1^), marking a decrease of up to 41.8%. In contrast, CF + BP and CF + 2BP had the highest EC values, while also having the lowest soil organic matter compared to CF. Soil CaCO_3_ was significantly increased with the use of CF compared to the rest of the treatments, excluding BP. The lowest P and Na contents were attained by BP, whereas K content was decreased under CF + OF and CF + BP. Finally, soil N content was unaffected by the experimental treatments.

Under supplementary fertigation, CF + 2BP/SF treatment yielded the highest soil pH (8.04), which was 2.3% higher than the lowest pH (7.73) under CF/SF. In contrast, CF + 2BP/SF resulted in the lowest soil EC (1.91 mS cm^−1^), demonstrating a 69.6% reduction compared to the highest EC elicited by CF + BP/SF (3.24 mS cm^−1^). The CF + BP/SF treatment, however, exhibited a reduction in soil organic matter. The same treatment increased CaCO_3_ compared to CF + 2BP/SF. The N content increased under CF + 2BP/SF, without differing significantly from CF/SF. BP/SF alone application had a reduced P content relative to CF + BP/SF and CF + 2BP/SF. However, the use of CF + BP/SF resulted in reduced soil K content, while both CF + OF/SF and BP/SF reduced the content of Na in soils.

The impacts of the examined treatments on the characteristics of leachates are presented in Table S5 of the Supplementary File. All examined parameters, with the exception of pH, were unaffected by the different treatments applied. However, the reduced pH, with or without supplementary fertigation, was attained with the CF (pH 7.21) and CF/SF (pH 7.41) applications relative to the other treatments, which reached pH values ranging from 7.54 to 7.71.

### 2.2. Plant Growth, Yield, and Physiological Parameters

Under no supplementary fertigation, during the intermediate stage (40 DAT), plants had the highest heights and leaf numbers with the use of CF + BP (Table 2). In contrast, the lowest plant heights were recorded with CF and CF + 2BP, while the plants’ leaf numbers were reduced with the application of BP alone compared to CF + BP. Both BP and CF + BP reduced stem thickness, while SPAD and chlorophyll fluorescence remained unaffected. At the final stage (84 DAT) of the experiment, stem thickness was reduced by up to 24.3% (base) and 30.4% (mid) with the use of BP. However, plant height, leaf number, SPAD, and chlorophyll fluorescence remained unaffected, averaging 188.5 cm, 17.2, 60.4, and 0.72, respectively. Interestingly, Chl a was decreased (up to 37.6%) by the CF + BP treatment, whereas Chl b, Total Chl and Total Car remained unaffected (Table S6).

Under supplementary fertigation, at 40 DAT, plant height was reduced under CF + OF/SF relative to the other treatments excluding BP/SF. The lowest leaf number was marked in plants grown in BP/SF treatment. Stem thickness was highest under CF + 2BP/SF. SPAD was increased under CF + OF/SF relative to BP/SF and CF + 2BP/SF, while chlorophyll fluorescence remained unaffected. At 84 DAT, base stem thickness was reduced under the BP/SF treatment up to 13.0%, while CF + BP/SF caused a reduction in chlorophyll fluorescence compared to CF/SF. In contrast, other growth-related indicators remained unaffected.

Tomato plant organ biomass was affected by the examined treatments (Table 3). Under no supplementary fertigation, both leaf and stem weight were reduced under BP, compared to CF + OF, corresponding to an overall reduction of 81.0% and 72.5%, respectively. Root DM was highest with the BP, while leaf DM was elevated with CF + 2B compared to CF + OF. Finally, the overall plant biomass production remained unaffected by the examined fertilization regimes.

Under supplementary fertigation, BP/SF produced significant decreases in the weight of leaves, stems, and overall biomass, compared to CF + 2BP/SF. However, the same treatment, and CF/SF, resulted in increased leaf DM compared to CF + OF/SF. In contrast, root DM remained unaffected.

Under no supplementary fertigation, no significant differences occurred among treatments in terms of plant fruit number, mean fruit weight, total fruit weight, and yield (Table 4). However, under supplementary fertigation, fruit number was increased (by up to 53.1%) with the use of CF + 2BP/SF, compared to CF + OF/SF and BP/SF. The same treatment also reported an increase in total fruit weight, corresponding to a 46.0% increase compared to the lowest value attained by BP/SF. In contrast, both mean fruit weight and marketable yield remained unaffected by the examined treatments (Table 4).

### 2.3. Plant Tissue Nutrient Content

**Figure 1:**
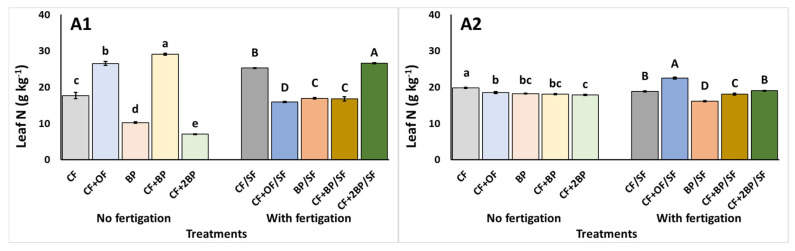

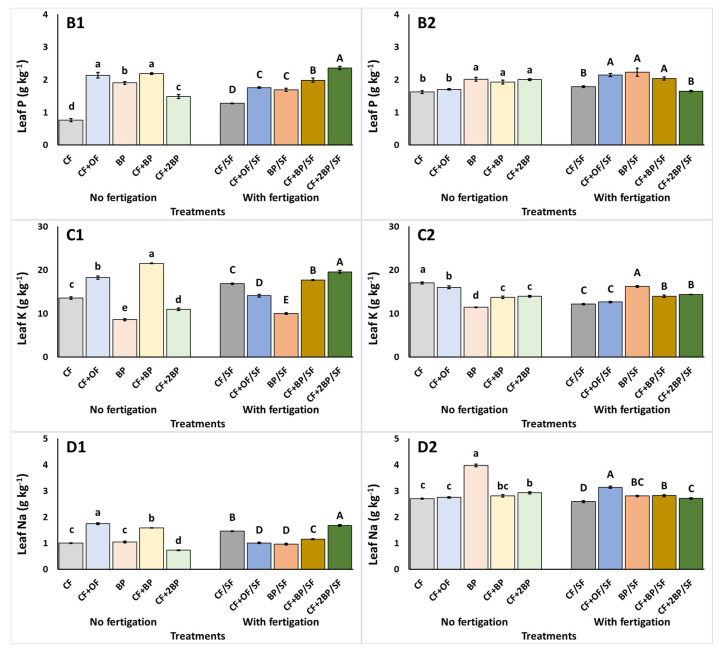
The effects of CF (conventional), CF + OF (conventional and organic) BP (bioproduct; 150 kg ha^−1^) CF + BP (conventional with bioproduct; 150 kg ha^−1^), CF + 2BP (conventional with bioproduct; 300 kg ha^−1^) basal fertilization, without or with supplementary NPK fertigation (SF) on leaf (**A1**,**A2**) nitrogen (N), (**B1**,**B2**) phosphorus (P), (**C1**,**C2**) potassium (K), and (**D1**,**D2**) sodium (Na) content (g kg^−1^ DW) at the (1) intermediate and (2) final stage of the experiment. Values represent mean (*n *= 5). Error bars show standard error. On columns, different lowercase letters (without supplementary fertigation) and uppercase letters (with supplementary fertigation-SF) indicate significant differences (*p* < 0.05).

At 40 DAT, under no supplementary fertigation, leaf N declined under CF + 2BP, but accumulated under CF + BP treatment. This was reflected by a 312.6% difference in the N content between the two treatments (Figure 1(A1)). In addition, leaf P was highest with both CF + BP and CF + OF, while a decline of up to 185.9% was observed under CF (Figure 1(B1)). The CF + BP treatment resulted in the highest leaf K content, with an increase of 149.95% compared to the BP treatment (Figure 1(C1)). Finally, leaf Na was highest with the use of CF + OF, while the use of CF + 2BP resulted in the lowest leaf Na (Figure 1(D1)). At 84 DAT, the highest leaf N content was observed under the CF application compared to the rest of the treatments. CF + 2BP resulted in a significant decline of N accumulation (up to 9.8%) (Figure 1(A2)). In contrast, all BP applications resulted in significant increases of leaf P content, compared to both CF and CF + OF (Figure 1(B2)). BP alone resulted in the lowest and highest K and Na contents, respectively. In contrast, CF resulted in the highest leaf K accumulation, while both CF and CF + OF resulted in the lowest leaf Na accumulation (Figure 1(C2,D2)).

Under supplementary fertigation, the highest macronutrient accumulation at 40 DAT was reported under CF + 2BP/SF. Conversely, the lowest macronutrient accumulation was observed under specific treatments, as leaf N content declined most under CF + OF/SF (Figure 1(A1)), P content under CF/SF (Figure 1(B1)), K content under BP/SF (Figure 1(C1)) and Na content under CF + OF/SF and BP/SF (Figure 1(D1)). The combination of CF and BP, especially under CF + 2BP/SF, resulted in greater leaf K accumulation (Figure 1(C1)). The final sampling revealed that the highest leaf N content was observed under the CF + OF/SF application, while the lowest occurred under BP/SF (Figure 1(A2)). Leaf P content was significantly decreased under both CF/SF and CF + 2BP/SF, compared to the rest of the treatments (Figure 1(B2)). Leaf K content was the highest with BP/SF, while it declined (up to 25.1%) under CF/SF and CF + OF/SF (Figure 1(C2)). Finally, the highest leaf Na content was observed under CF + OF/SF, while the lowest was observed under CF/SF (Figure 1(D2)).

**Figure 2:**
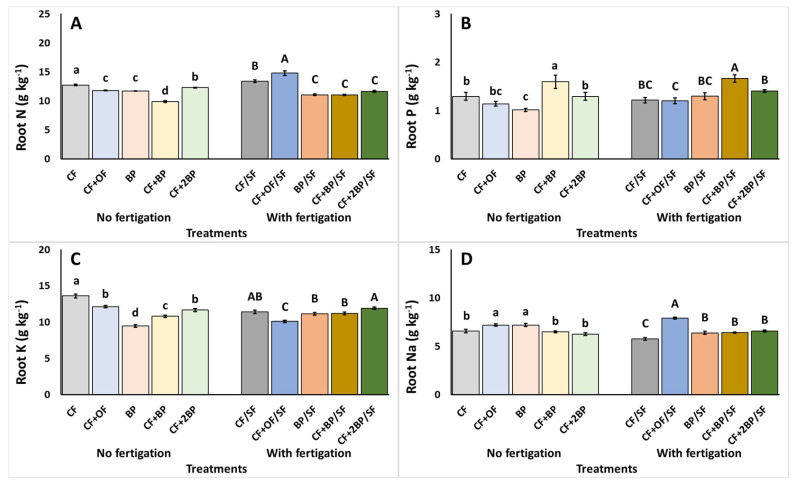
The effects of CF (conventional), CF + OF (conventional and organic) BP (bioproduct; 150 kg ha^−1^) CF + BP (conventional with bioproduct; 150 kg ha^−1^), CF + 2BP (conventional with bioproduct; 300 kg ha^−1^) basal fertilization, without or with supplementary NPK fertigation (SF) on root (**A**) nitrogen (N), (**B**) phosphorus (P), (**C**) potassium (K), and (**D**) sodium (Na) content (g kg^−1^ DW) collected after the cultivation period. Values represent mean (*n *= 5). Error bars show standard error. On columns, different lowercase letters (without supplementary fertigation) and uppercase letters (with supplementary fertigation-SF) indicate significant differences (*p* < 0.05).

Figure 2 shows the macronutrient content of tomato plant roots at 84 DAT. Under no supplementary fertigation, CF + BP application resulted in the lowest root N accumulation, corresponding to a reduction of 22.3% relative to the highest value observed under CF (Figure 2A). In contrast, CF + BP resulted in the highest root P content, while BP-alone application caused a 36.5% reduction, attaining the lowest root P content (Figure 2B). Root K was also the lowest with the BP-alone application, representing a 30.4% decrease over the highest value attained by CF (Figure 2C). Finally, root Na content was the highest under the CF + OF and BP treatments compared to the rest of the treatments (Figure 2D).

Under supplementary fertigation, root N content was significantly enhanced under CF + OF/SF but decreased with BP applications (Figure 2A). Root P content followed a similar trend under no supplementary fertigation, as CF + BP/SF resulted in the highest accumulation (Figure 2B). Additionally, root K content was the highest under CF + 2BP/SF, without differing significantly relative to CF/SF. In contrast, CF + OF/SF caused a decrease in root K content of up to 15.2% (Figure 2C). Finally, the highest root Na content was recorded for the CF + OF/SF treatment (Figure 2D).

**Figure 3:**
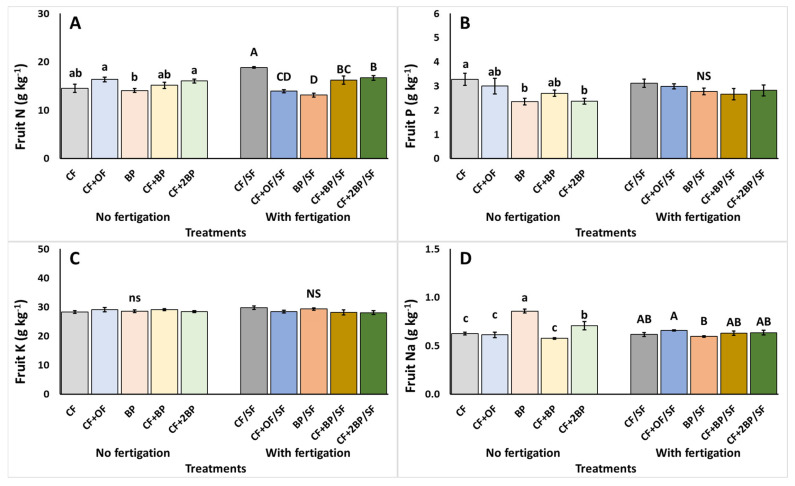
The effects of CF (conventional), CF + OF (conventional and organic) BP (bioproduct; 150 kg ha^−1^) CF + BP (conventional with bioproduct; 150 kg ha^−1^), CF + 2BP (conventional with bioproduct; 300 kg ha^−1^) basal fertilization, without or with supplementary NPK fertigation (SF) on fruit (**A**) nitrogen (N), (**B**) phosphorus (P), (**C**) potassium (K), and (**D**) sodium (Na) content (g kg^−1^ DW). Values represent mean (*n *= 7). Error bars show standard error. On the columns, different lowercase letters (without supplementary fertigation) and uppercase letters (with supplementary fertigation-SF) indicate significant differences (*p* < 0.05), while ns or NS indicate no significant differences.

Mineral accumulation in fruits is examined in Figure 3. Under no supplementary fertigation, fruit N content was significantly reduced (up to 14.0%) under BP, compared to CF + OF and CF + 2BP (Figure 3A). The phosphorus content of the fruit was reduced under the BP and CF + 2BP applications compared to CF (Figure 3B). Conversely, the highest Na content in the fruit was detected under the BP treatment (Figure 3D). Finally, the fruit’s K content remained unaffected by the experiment applications (Figure 3C).

Under supplementary fertigation, the highest fruit N content was observed under CF/SF application, while the lowest fruit N content was observed under BP/SF (Figure 3A). Similarly, a significant increase in fruit Na content occurred under BP/S, compared to CF + OF/SF, corresponding to a decrease of 9.4% (Figure 3D). In contrast, both the P and K contents in fruit remained unaffected by the examined treatments (Figure 3B,C).

### 2.4. Total Phenols Content, Antioxidant Activity, and Stress Indicators

**Figure 4:**
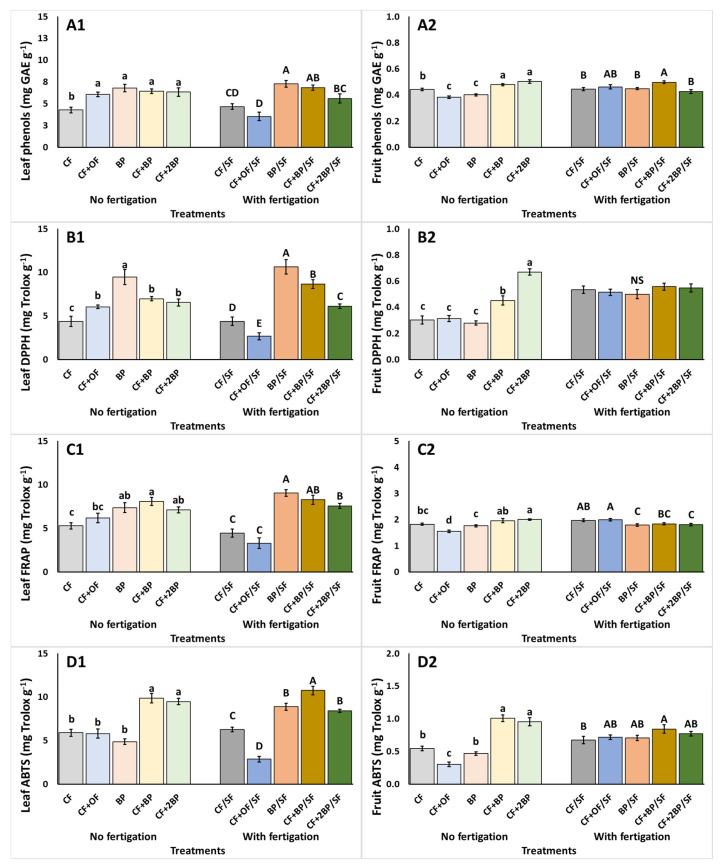
The effects of CF (conventional), CF + OF (conventional and organic) BP (bioproduct; 150 kg ha^−1^) CF + BP (conventional with bioproduct; 150 kg ha^−1^), CF + 2BP (conventional with bioproduct; 300 kg ha^−1^) basal fertilization, without or with supplementary NPK fertigation (SF) on (**A1**,**A2**) total phenolic content (mg GAE g^−1^ FW), (**B1**,**B2**) DPPH (mg Trolox g^−1^ FW), (**C1**,**C2**) FRAP (mg Trolox g^−1^ FW), (**D1**,**D2**), and ABTS (mg Trolox g^−1^ FW) of leaves (1) and fruit (2). Values represent mean (*n *= 5 for leaves; *n *= 7 for fruits). Error bars show standard error. On columns, different lowercase letters (without supplementary fertigation) and uppercase letters (with supplementary fertigation-SF) indicate significant differences (*p* < 0.05), while NS indicates no significant differences.

Under no supplementary fertigation, decreased leaf total phenolics were observed under CF (Figure 4(A1)). Similarly, CF resulted in the lowest DPPH, while an increase (up to 117%) was observed using BP (Figure 4(B1)). The CF + BP treatment resulted in a significant increase of FRAP, compared to CF and CF + OF (Figure 4(C1)), while leaf ABTS was enhanced under CF + BP and CF + 2BP (Figure 4(D1)). In the fruit, total phenolics declined (up to 13.3%) under CF + OF and BP compared to CF, whereas the combined treatments of CF + BP and CF + 2BP induced an increase (Figure 4(A2)). Additionally, CF + 2BP resulted in increased (up to 140%) DPPH in fruit. The lowest values were observed with CF, CF + OF, and BP (Figure 4(B2)). The CF + OF treatment resulted in the lowest values of FRAP and ABTS in fruit, while the highest values were obtained with the combination of CF and BP (CF + BP, CF + 2BP) (Figure 4(C2,D2)).

Under supplementary fertigation, the total phenols and FRAP of leaves were enhanced under BP treatments compared to CF/SF and CF + OF/SF (Figure 4(A1,C1)). In addition, BP/SF exhibited the highest leaf DPPH and ABTS, showing respective increases of 301.2% and 274.8%. The lowest values were attained under CF + OF/SF (Figure 4(B1,D1)). The tomato fruit total phenolic contents were significantly higher with the application of CF + BP/SF relative to the other treatments, excluding CF + OF/BP (Figure 4(A2)). Additionally, a significant reduction in fruit FRAP occurred under BP/SF (up to 10.6%) compared to CF/SF and CF + OF/SF (Figure 4(C2)). In addition, fruit ABTS was significantly increased by the application of CF + BP/SF relative to CF/SF (Figure 4(D2)). Finally, the fruit’s DPPH did not differ significantly among treatments (Figure 4(B2)).

**Figure 5:**
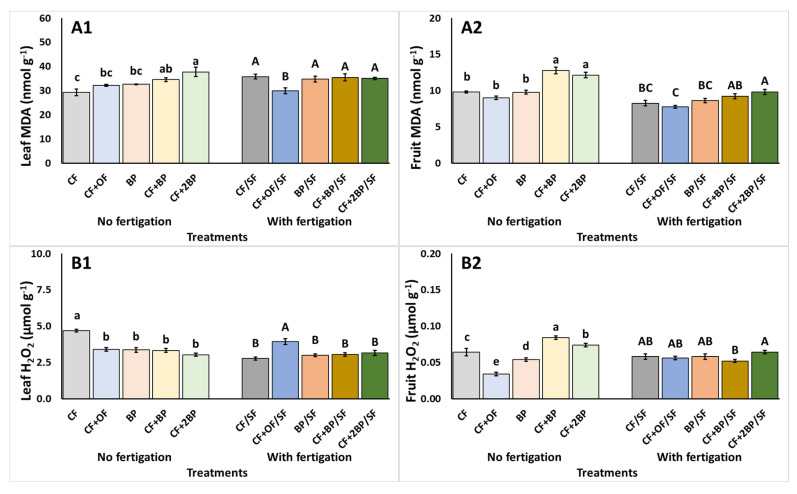
The effects of CF (conventional), CF + OF (conventional and organic) BP (bioproduct; 150 kg ha^−1^) CF + BP (conventional with bioproduct; 150 kg ha^−1^), CF + 2BP (conventional with bioproduct; 300 kg ha^−1^) basal fertilization, without or with supplementary NPK fertigation (SF) on (**A1**,**A2**) Malondialdehyde (MDA) content (nmol g^−1^ FW) and (**B1**,**B2**) Hydrogen peroxide- μmol g^−1^) of leaves (1) and fruit (2). Values represent mean (*n *= 5 for leaves; *n *= 7 for fruits). Error bars show standard error. On columns, different lowercase letters (without supplementary fertigation) and uppercase letters (with supplementary fertigation-SF) indicate significant differences (*p* < 0.05).

The effect of the applied treatments on the stress response of plants, as indicated by MDA and H_2_O_2_, are presented in Figure 5. Under no supplementary fertigation, compared to CF, lipid peroxidation in terms of MDA in leaves was enhanced under CF + BP and CF + 2BP (Figure 5(A1)). In addition, plants treated with CF + BP and CF + 2BP showed an elevated fruit MDA compared to the rest of the treatments (Figure 5(A2)). The highest leaf H_2_O_2_ was presented under the CF treatments, compared to the rest of the treatments (Figure 5(B1)). Moreover, the lowest value of H_2_O_2_ in fruit was recorded under the CF + OF treatment, whereas CF + BP exhibited the highest H_2_O_2_, culminating in an increase of up to 147.1% (Figure 5(B2)).

Under supplementary fertigation, the use of CF + OF/SF resulted in decreased (up to 44.4%) MDA and increased (up to 42.4%) H_2_O_2_ compared to the rest of the treatments (Figure 5(A1,B1)). The fruit MDA was significantly lower with CF + OF/SF, compared to both CF + BP/SF and CF + 2BP/SF. In contrast, the highest MDA was recorded under CF + 2BP/SF, which produced an increase of up to 34.8% (Figure 5(A2)). Finally, fruit H_2_O_2_ levels were reduced (up to 18.8%) with CF + BP/SF compared to CF + 2BP/SF (Figure 5(B2)).

### 2.5. Fruit Quality Assessment and Macroscopic Evaluation

Fruit color indices are presented in Table S7 of the Supplementary Material. Most indices, including lightness (L* value), redness (a* value), b* value, hue, color index, whiteness index, and browning index, remained unaffected by the examined treatments. However, chroma value was significantly affected by the treatments, either with or without supplementary fertigation. Under no supplementary fertigation, chroma value decreased (up to 8.0%) under CF, compared to the value attained by CF + BP. Under supplementary fertigation, however, chroma value declined by 7.2% relative to the application of CF + BP/S.

Selected fruit quality parameters are presented in Table 5. Fruit firmness was only affected under supplementary fertigation, as decreases occurred with the application of CF + OF/SF and CF + 2BP/SF, relative to CF + BP/SF application, which enhanced firmness by up to 71.2% (Table 5). Under no supplementary fertigation, the total soluble solids (TSS) of tomato fruits were higher with the application of CF + BP, relative to the other treatments. In contrast, BP use saw a decline in fruit TSS of up to 23.4%. Additionally, under supplementary fertigation, both CF + OF/SF and BP/SF resulted in a significant decline in TSS relative to the other treatments, excluding CF + 2BP/SF. However, titratable acidity (TA) and sweetness/ripening index (TSS/TA) remained unaffected by the examined applications. Both lycopene and *β*-carotene were influenced by the examined treatments only under no supplementary fertigation; no significant changes occurred under supplementary fertigation. More specifically, fruit lycopene was increased under CF + BP compared to the rest of the treatments, with the exception of CF + OF. This culminated in an increase of lycopene by up to 38.5%. The use of BP alone decreased (up to 16.9%) *β*-carotene in relation to the other treatments. In contrast, the highest *β*-carotene in fruit was a result of the CF + 2BP treatment. Fruit ascorbic acid under no supplementary fertigation declined with the use of CF + OF and CF + BP, compared to CF and CF + 2BP. However, under supplementary fertigation, it was enhanced with CF + BP/SF and CF + 2BP/SF, in relation to the other treatments.

Macroscopic evaluations of tomato fruits are also presented in Table 5. Under no supplementary fertigation, fruit aroma was markedly improved with the use of CF compared to the rest of the treatments, excluding CF + OF. In contrast, the use of BP resulted in the lowest aroma score, with a decrease of up to 11.5%. Under no supplementary fertigation, aroma marginally declined under CF + OF/SF, compared to CF/SF. Finally, the appearance score significantly increased with CF + OF in relation to the scores attained by both BP and CF + 2BP, under no supplementary fertigation. Finally, marketability was not influenced by the treatments under examination.

Finally, tomato fruit symptoms, including blossom end rot, cracking, insect attack, russeting, and wounding, as examined in Table S8 of the Supplementary File, generally did not differ significantly among the various treatments.

## 3. Discussion

Soluble bio-based substances produced through chemical hydrolysis can be utilized to produce value-added fertilizers. These substances can be applied to promote plant growth and used as alternatives to commercial mineral or organic N fertilizers [19]. The application of BPs has yielded increased productivity in various crops [21,24]. However, the specific mode of action that can improve plant growth and yield has not been clearly demonstrated [18,19]. In addition, most studies have focused on primarily assessing BPs for their input on yield and quality, while neglecting to assess the effects on the bioactive substances and antioxidant activities. This overlooks the potential impact of the nutrient availability and biochemical characteristics of these materials on compounds such as phenolics, antioxidants, and sugars [25]. In the current study, BP was applied to greenhouse tomato cultivation alone or with conventional fertilizers at two BP rates. This was conducted to elucidate the impact of the BP in crop production, while effectively understanding its direct input when applied under a typical basal fertilization scheme. In addition, the experiment examined its potential in comparison to a commercial organic (vinasse-based) soil amendment to identify divergent factors such as solubility.

### 3.1. Soil Physicochemical Parameters and Leachates Characterization

Bioproducts from anaerobic digestates exhibit variations in their chemical attributes, as they depend on the feedstock used during their production. These variations, which include soil organic matter, soil organic carbon, N content, and C/N ratio, influence their impact on soil properties [18,26]. In the current study, under no supplementary fertigation, the application of BP alone resulted in increased pH and decreased EC compared to the other treatments, at the end of the experiment. The decreased EC was associated with decreases in the soil P and Na content relative to the other treatments. In contrast, the EC in the CF + BP and CF + 2BP treatments was increased, and was correlated with decreases in the soil organic matter content. This is related to the composition of the applied BP material, which is characterized by a richness of mineral elements [18]. Therefore, when combined with conventional fertilizers, BPs can increase the soil EC, as they introduce soluble ions [27]. The use of BPs as potent biostimulants can also have a beneficial effect on the microbial community of the soil, which may have intensified soil organic matter decomposition [28,29]. This in effect accelerates the mineralization of soil organic matter, effectively increasing the soil EC [30], and this can be a possible explanation of the increased EC observed under CF + BP applications in the present study.

### 3.2. Plant Growth, Yield, and Physiological Parameters

Previous research has indicated the potential of BPs as soil amendments, biofertilizers, and biostimulants, with the capacity of improving the growth of various plants [18]. However, the method of application varies among studies, and includes foliar applications and supply in the root zone with direct basal dressing or fertigation applications [31]. A critical factor in the agronomic potential of BPs is their high solubility, which may improve plant nutrient availability and uptake [19]. Thus, the current study supplied the BP directly to the root zone as a means of improving its efficiency in distribution and dosing [31].

In the current study, there was a significant influence of the treatments examined on the growth of tomato plants, with distinct variations based on the growth stage of the plants and the use of supplementary fertigation. More specifically, at the intermediate stage, plants treated solely with BP exhibited comparable plant height, leaf number, SPAD, and F_v_/F_m_ to those treated with CF, while the use of CF + BP resulted in significantly higher plant height. However, a decline in stem thickness occurred under BP and CF + BP, which was alleviated by the application of CF + 2BP. Similar results were attained under supplementary fertigation, with a significant improvement in plant height and leaf number occurring with CF + BP/SF and CF + 2BP/SF compared to CF + OF/SF and BP/SF, respectively. As previously reported by Sortino et al. [20], the influence of BP can be evidenced early during the cultivation period. However, at the end of the cultivation period, plant growth-related parameters as well as biomass production and fruit yield were unaffected by the inclusion of BP compared to mineral fertilization. Although growth parameters were consistently higher with the use of CF compared to BP-alone application, the differences were not statistically significant, likely due to the variability in plant responses rather than sample size, which was adequate (*n *= 9). Given this, the refinement of BP dosage or application frequency may accentuate its biostimulant effects. These results contrast previous findings, as growth was stimulated with the inclusion of BP in various crops (e.g., tomato, common bean) [20,22]. This has been previously observed with the application of humic and humic-like substances [32,33]. In a subsequent study, Fascella et al. [24] reported a significant enhancement of orange jasmine (*Murraya paniculata* L. Jacq.) plant height, number of leaves, and biomass production. Interestingly, the combined use of CF and BP did not significantly improve growth-related parameters, compared to the use of CF alone. This is reflected in tomato fruit yield as well, which was unaffected by the examined parameters. The findings of this study align to those reported by Barzee et al. [34], as the application of anaerobically digested food waste and dairy manure on field-cultivated tomato (*Lycopersicon esculentum* Mill.), compared to mineral fertilization, resulted in comparable SPAD and fresh tomato yield. However, Bilalis et al. [35] observed decreased fruit number, fruit weight, and yield with the application of seaweed compost fertilization compared to conventional inorganic fertilization. A likely explanation is that the availability of nutrients either in soil or under CF application was already sufficient to maintain plant growth, with any BP contributions being less apparent. In addition, the effects of BP may be influenced by factors such as plant developmental stage, cultivation conditions, and cultivation duration. Interestingly, under supplementary fertigation, the application of CF + 2BP/SF promoted greater leaf, plant, and fruit weight relative to BP/SF, while also enhancing fruit number and stem weight when compared to both BP/SF and CF + OF/SF.

### 3.3. Plant Tissue Nutrient Content

The macronutrient content of tomato plants was significantly influenced by the examined applications during both sampling periods (40 and 84 DAT). More specifically, at 40 DAT, plants exhibited decreased macronutrient accumulation with the application of BP alone compared to CF. This was previously observed by Fragalà et al. [13], as the sole application of BP at the same base rate employed in current study (150 kg ha^−1^) resulted in decreased N accumulation in lettuce (*L. sativa* L.) leaves. However, when the researchers combined BP (150 kg ha^−1^) with full mineral fertilization, an increase in N accumulation was observed relative to mineral fertilization alone. This was attributed to the enhancement of NUE by the use of BP, potentially due to a higher nitrate reductase activity during early growth as well as a significant reduction of N leaching [13]. Consistent with these findings, seaweed extract-based fertilizers have a role in the initial assimilation of N in plants due to the upregulation of enzymes such as nitrate reductase, glutamine synthetase and glutamate synthase [36,37]. The same results were indicated in the current study, as CF + BP increased the intermediate N content in leaves. In addition, the same treatment resulted in enhanced P, K, and Na content of leaves relative to CF. However, CF + 2BP resulted in decreased N and K accumulation in comparison to CF. This may be associated with excess ammonia volatilization and increased N-immobilization, which may cause N deficiency as well as elevated soil EC, which may have influenced plant nutrient uptake [38,39]. Contrasting this, under supplementary fertigation, CF + 2BP resulted in elevated intermediate leaf nutrient accumulation. The increased uptake may be related to the increased availability of nutrients provided by the sustained fertigation applications coupled with the elevated solubility of BPs, which may lead to improvements in nutrient availability and uptake by plants [19]. At the final sampling period, under no supplementary fertigation, the CF treatment resulted in the highest N and K content in leaves and roots. In contrast, the use of BP, with or without CF, resulted in decreases of N content. This corresponds with decreased nitrate reductase activity during later plant growth stages, as suggested previously [13]. A similar condition was observed with K content, suggesting decreased K uptake in the tomato plants. Previous reports have associated the use of humic substances with increases in the activity of root H^+^-ATPase, which affects nutrient uptake [40]. These effects may also result from nutrient imbalances caused by using BP, such as imbalanced N/K ratios and excessive N, which can aggravate K deficiency while also impairing N uptake [41]. Additionally, tomato fruit under no supplementary fertigation exhibited decreased P content with the use of BP, relative to CF. Although this was ameliorated with the use of CF + BP, these results may be linked to the intrinsic characteristics of the BPs, in which P is often present in less readily available forms [42]. In contrast, the general use of BP under no fertigation resulted in the enhancement of P uptake in leaves and roots, as similarly observed by Foughar et al. [43]. These results may be associated with the late P availability provided by BP, as well as imbalanced nutrition [44]. Interestingly, the application of artificial humic substances can result in increased P fixation in soil, limiting its availability to plants [45]. In addition, the underlying K deficiency may have negatively influenced the translocation of P from leaves to fruit [46]. Contrasting these results, Massa et al. [23] reported that humic-like substances can enhance P availability by preventing calcium phosphate precipitation. Under supplementary fertigation, however, plants exhibited increased leaf K contents with the application of BP, especially under the BP/SF treatment. Leaf P accumulation followed a similar pattern, except for the CF + 2BP/SF treatment. In contrast, leaf N content was reduced under BP/SF and CF + BP/SF. However, the study of Sortino et al. [20] reported that compared to control, the bio-organic fraction did not cause any significant impacts on leaf macronutrient content (e.g., N, P, K, Na). These differences may be associated with the variation of BP solubility, which is of particular importance and might affect nutrient availability.

### 3.4. Total Phenols Content, Antioxidant Activity, and Stress Indicators

The phenolic profile and antioxidant activity in tomato leaves and fruit exhibited distinct variations in response to the different fertilization applications. Regardless of fertigation application, leaves exhibited elevated total phenolic contents under the BP treatments relative to CF. This underscores the influence of BP on the plants’ metabolic processes and the stimulation of the secondary metabolism [18]. A similar response was observed in fruit, under no supplementary fertigation, where the application of BP, particularly in combination with CF, resulted in enhanced phenolic content as well as increased antioxidant activity, reflected by the increases of DPPH, FRAP, and ABTS. This finding is significant, as enhancing the antioxidant activity in fruit is particularly desirable, given the positive effects for human health and nutritional quality [47]. Comparable results were obtained by Laužikė et al. [48] for tomatoes, as the introduction of digestates resulted in accumulation of antioxidant compounds. Similarly, the utilization of by-products from livestock waste augmented the presence of bioactive compounds, particularly enhancing fruit phenolic compounds and antioxidant activity [25]. The positive effect on the plants’ secondary metabolism, as indicated by the enhancement of phenolic compounds may have be related to the regulation of the phenylpropanoid pathways [49]. However, the influence of biowaste-derived substances on fruit health-related compounds depends on the composition and concentration of the substance used, plant genotype, environmental conditions, and cultivation practices [50].

The observed effects of the examined applications on total phenolics and antioxidant activities were accompanied by a marginal increase in leaf MDA and a corresponding decrease in leaf H_2_O_2_, with the application of BP under no supplementary fertigation. The decreased leaf H_2_O_2_ suggests that the plant’s antioxidant mechanism is actively scavenging reactive oxygen species, reflected by the enhanced secondary metabolism [51]. However, enhanced lipid peroxidation suggests prior cellular membrane damage induced by oxidative stress [52]. These observations are accompanied by increases in fruit MDA and H_2_O_2_, likely due to the stimulation of the antioxidant mechanism prompted by declining nutrient availability [23]. Interestingly, humic substances have been shown to elicit stress responses due to the activation of hormonal and molecular pathways associated with stress adaptation [40,53]. The utilization of organic fertilizers has also been associated with increases in lipid peroxidation and the accumulation of nutritional compounds including phenolics as a consequence of stress induced by limited nutrient availability. This is further supported by the addition of supplementary fertigation, which elicited no significant alterations in leaf MDA and H_2_O_2_ across CF and BP treatments.

### 3.5. Fruit Quality Assessment and Macroscopic Evaluation

Tomato fruit quality involves a number of measurable physico-chemical parameters beyond physical appearance [20]. The use of substances derived from biowaste has been shown to influence tomato quality by enhancing sensory attributes and nutritional value associated with influences in produce quality [18]. Lycopene, as a major carotenoid in tomato fruits, presents significant antioxidant activity against chronic diseases. Lycopene is influenced by a range of genetic and environmental factors, including the cultivation conditions and fertilizers applied [50]. In the current study, lycopene was positively influenced by CF + BP, exhibiting a 27.2% increase relative to CF. Similar findings were observed with the use of digestates obtained by animal manures and plant waste and legume-derived hydrolysates [54,55]. Interestingly, earlier research has identified a strong correlation between the lycopene levels in fruit and their antioxidant activity [25]. In contrast, Abou Chehade et al. [49] demonstrated that the use of various food processing by-products in the cultivation of tomatoes (*S. lycopersicum* L.) with uniform organic fertilization presented no alterations in fruit lycopene. Similar results were obtained by Morra et al. [56] with the use of a slurry-derived digestate. This discrepancy may be associated with the cultivation methods applied and the rates of the examined substances as well as their composition. The TSS content is of significant importance in the tomato processing industry, as its improvement can enhance product output and reduce processing costs [50]. In the current study, the use of BP under no supplementary fertigation resulted in a decrease of TSS. The content of *β*-carotene had a similar fate, being reduced under the BP-alone application. Similarly, Alan et al. [50] reported a decline of TSS with the use of digestates, compared to conventional fertilization. However, CF + BP resulted in an increase of TSS, and CF + 2BP in an increase of *β*-carotene compared to CF, whereas the introduction of supplementary fertigation nullified these differences. These positive effects with the utilization of biowaste-derived substances in the cultivation of tomato plants has been previously noted [25,34].

### 3.6. Final Considerations and Future Perspectives

Taken together, the results of the current study show that BP-alone application may not adequately address the nutritional requirements of tomato plants, impacting nutrient uptake and quality attributes but not significantly affecting growth and productivity. This response is likely explained by the observation that, despite containing all of the mineral nutrients needed by plants, these materials alone cannot fulfill the plants’ nutritional needs, especially for plants with an extended growing period [13,18]. Consequently, mineral fertilization remains necessary for achieving adequate nutrient availability, in the form of basal fertilization and/or fertigation during the cultivation. However, BP-alone application could be improved by increasing the application rates or conducting successive applications. However, the current study indicates that even though plant growth or productivity remained relatively unaffected, BP can lead to enhancements in produce quality, as indicated previously [31,40]. However, BP application was associated with the induction of stress responses and a subsequent increase in antioxidant activity, likely mediated by the activation of stress-related signaling pathways [53]. A significant aspect of the prevalent effects of BPs may be related to the application method, dose, frequency, as well as environmental conditions and the plant species under examination. Nevertheless, as observed in the current study, BPs can enhance produce quality by promoting physiological and metabolic functions, and thus can be potentially utilized as biostimulants as supplements to conventional fertilization. Besides, the production of BPs is relatively low cost (0.1–0.5 EUR kg^−1^), and environmentally sustainable, as production does not cause a depletion of soil organic matter or fossil deposits [18]. The adaptation of BPs is also expected to comply with the regulatory framework of the European Union, with their utilization as alternatives and supplements to conventional fertilization adhering to the European common agricultural policy [13,57]. In addition, at a commercial scale, agricultural use of BPs could improve quality and production at a relatively low cost, without causing ecological harm [22]. However, future research should focus on optimizing the efficacy of BP application through adjustments in application rate and frequency, while also assessing across diverse plant species and cultivation systems.

## 4. Materials and Methods

### 4.1. Plant Material and Experimental Site

A pot study was carried out at the experimental greenhouse of the Cyprus University of Technology, Limassol, Cyprus, from March to June 2023, with a total duration of 84 days after transplanting (DAT). Table S1 shows the soil properties used in the pot experiment.

The study was conducted to evaluate the potential role and use of a BP material as a fertilizer component in the production of tomato (*S. lycopersicum* cv. ‘Elpida’). The BP material was derived from urban waste; the hydrolysis process was undertaken by ACEA Pinerolese Industriale S.p.A (Turin, Italy), as directed by HYSYTECH S.r.l. (Turin, Italy) [18]. The hydrolysis and sedimentation process as well as BP production was described in the details of previous work [58]. The characterization of the BP is presented in Table S2. To assess its performance, five different basal fertilization regimens were applied per treatment; (1) conventional fertilization (CF), (2) conventional and organic fertilization (CF + OF), (3) BP at 150 kg ha^−1^ (BP), 4) conventional fertilization and BP at 150 kg ha^−1^ (CF + BP), and (5) conventional fertilization and BP at 300 kg ha^−1^ (CF + 2BP). The treatments are presented in Table S3 and fertilization applications are presented in Table S4. The conventional fertilizers consisted of ammonium nitrate (NH_4_NO_3_), triple superphosphate (TSP) and potassium nitrate (KNO_3_). For organic fertilization, a commercial vinasse-based organic fertilizer (Phenix 6-8-15, Hello Nature International S.r.l, Biandrate, Italy) was used. All fertilizers were thoroughly mixed with the soil. To diversify the experiment and examine the long-term impact of basal fertilization, the base treatments were duplicated, and the second batch of plants received supplementary fertigation (denoted as SF), as described below.

Plastic pots (9.5 L, 24 × 24 cm) were filled with the prepared soil mixtures, in which tomato seedlings procured from a local nursery were transplanted at the stage of 3–4 true leaves. A total of 102 plants were used, twelve of which served as border plants. The plants were handled on a string according to the single pruning scheme, where the main stem grew vertically, and all lateral shoots were removed during the cultivation period. Plants were irrigated using a drip system, with each pot having one dripper. Irrigation frequency and time were based on crop growth stage; plants in the initial growth stages were irrigated once per day for 5 min, whereas at late stages plants were irrigated once per day for 10 min. Fertigation for SF treatments was conducted with solutions (EC: 1.5–2.5 mS cm^−1^; 500 mL per plant) made up of commercial fertilizers (i.e., 20-20-20) four times during the experiment, at an interval of approximately two weeks (31, 44, 56, and 70 DAT). The fertigation solution was applied manually at the surface of the soil in addition to routine irrigation practices. The interval of fertigation application followed common fertigation practices, typically occurring every one to two weeks. Plant protection products were applied following common agricultural practices. Temperature and relative humidity measurements were logged by meteorological stations. During the cultivation period, the temperature (°C) and relative humidity (%) fluctuation during day and night for the cultivation period, as presented in Figure S1, averaged 28.08 °C and 19.91 °C and 46.57% and 63.23%, respectively, during March through June 2023.

The experiment included ten treatments, with five basal fertilization applications and two fertigation applications. Each treatment consisted of 9 replicated plants randomly distributed in sub-plots. The experiment was carried out as a Randomized Complete Block Design.

### 4.2. Soil Physicochemical Parameters and Leachates Characterization

Soil samples were collected from 5 pots per treatment following the conclusion of the experiment. The collection was in the vicinity of plant roots. Soil samples were transported to the laboratory, where they were dried and hand-sieved to pass a 2 mm mesh. Calcium carbonate (CaCO_3_) was determined using Bernard calcimetry, and results were expressed as a percentage [59]. Soil organic matter was determined using the Walkley–Black volumetric method, and results were expressed as a percentage. Electrical conductivity (EC; mS cm^−1^) and pH were determined using saturation paste extracts with the FiveEasy Plus EC meter (Mettler Toledo International Inc., Greifensee, Switzerland) and HI 2211 pH meter (Hanna Instruments, Smithfield, RI, USA). Soil extraction was performed according to the ammonium acetate method, and then the determination of soil potassium (K) and sodium (Na) was performed by using flame photometry (Lasany Model 1832, Lasany International, Panchkula, India). Following extraction, P was determined using the Olsen sodium bicarbonate method spectrophotometrically. Soil nitrogen (N) content was measured with the Kjeldahl method (Digest automat K-439 and Distillation Kjelflex K-360, BUCHI Labortechnik AG, Flawil, Switzerland). Soil mineral contents were expressed as g per kg of dry weight (g kg^−1^ DW) [60].

Drainage samples were collected by five pots per treatment at the end of the experiment. Sampling was conducted following irrigation of the pots, where drainage was collected using saucers beneath the pots. Following sample filtration, the drainage solutions’ EC and pH were measured as described above. Subsequently, P was determined spectrophotometrically using the vanadate/molybdate assay (yellow method), and K and Na content were quantified using flame photometry (Lasany Model 1832, Lasany International, Panchkula, India). Mineral concentrations were expressed as grams per liter.

### 4.3. Plant Growth and Physiological Parameters

Plant growth-related indices were assessed at two points during the experiment; at an intermediate stage (40 DAT), and a final stage (84 DAT) for all plants per treatment. Parameters included plant height (cm), average leaf number, and stem thickness (base at 5 cm above ground, and mid at 1 m above ground). In addition, leaf chlorophyll fluorescence (F_v_/F_m_) and SPAD (soil plant analysis development) were also measured in all plants per treatment (*n*= 7–9 replicates/treatment). Chlorophyll fluorescence was measured using an OptiSci OS-30p Chlorophyll Fluorometer (Opti-Sciences Inc., Hudson, NH, USA), following incubation of the leaves in the dark for 15 min. SPAD was measured using a SPAD-502PLUS (Konica Minolta Inc., Osaka, Japan). The total fresh weight (FW; g) of leaves and stems was recorded at the experiment’s completion. Leaves removed (pruned) during de-leafing were also counted and weighted.

Fresh leaf tissue (five replications/treatment; 0.15 g) was used for chlorophylls extraction, with the addition of 7 mL methanol 100% and incubation in the dark. Extracts were then measured at 470, 653, and 666 nm. Finally, photosynthetic pigments, i.e., chlorophyll a (Chl a), chlorophyll b (Chl b), total chlorophylls (Total Chl) and total carotenoids (Total Car) contents were calculated [61]. Results were expressed as milligrams per gram of fresh weight (mg g^−1^ FW).

### 4.4. Produce Harvesting

The harvesting of tomato fruits (1360 fruits in total from the first 6 clusters of each plant) was carried out over a period of 35 days every five days, during which seven harvests were performed. Fruits were collected from all clusters at the pink–light red stage, and all harvested fruits were immediately counted and weighed. Yield was expressed as kg plant^−1^. During harvesting, fruits were assessed for symptoms of blossom end rot (BER), cracking, insect attack, russeting, and wounding, with results expressed in percentage. For chemical analyses, seven to nine fruits were selected from the fourth cluster, depending on the fruit setting. Assessment preceded storage for 24 h at room temperature. At least seven biological replicates were used.

### 4.5. Plant Tissue Nutrient Content

Plant tissue samples were collected and used for mineral analysis. Leaf tissue samples were collected twice, at 40 DAT (intermediate sampling) and at 84 DAT (final sampling). Root and fruit tissues were sampled at the end of the experiment. Each treatment consisted of five replicates/measurements taken from pooled tissue. Samples were initially dried at 52 °C for 4 d to attain constant weight. Dried samples were then weighted and ground. The leaf nitrogen (N) content was analyzed with the Kjeldahl method (Digest automat K-439 and Distillation Kjelflex K-360, BUCHI Labortechnik AG, Flawil, Switzerland). Dried tissue samples were then ashed at 550 °C for 270 min, and ash samples were digested using HCl 2 N. Extracts were then used for the determination of P spectrophotometrically (vanadate/molybdate assay -yellow method), and K and Na by flame photometry (Lasany Model 1832, Lasany International, Panchkula, India). Data were expressed as grams per kilogram of dry weight (g kg^−1^ DW).

### 4.6. Leaf and Fruit Total Phenols Content, Antioxidant Activity, and Stress Indicators

Tissue samples were extracted for the determination of the total phenols content and the antioxidant activity. Plant tissue samples were obtained from both leaves (0.5 g per replication, *n* = 5) and fruits (2 g per replication, *n *= 7), and stored at −20 °C. Following milling with methanol 50% and homogenization using the ULTRA-TURRAX T 25 mixer (IKA-Werke GmbH and Co., Staufen im Breisgau, Germany) for 60 s, samples were incubated in a sonication bath for 30 min, shaken (200 rpm) for 1 h, and centrifuged for (4 °C, 5000 rpm) for 15 min. The supernatant was then collected for further analysis.

Methanolic tissue extracts were used for the total phenolics content determination, utilizing the Folin–Ciocalteu method [62] with measurements at 755 nm. Results were calculated based on a gallic acid equivalents (GAE) calibration curve and expressed as mg GAE g^−1^ FW.

To evaluate the antioxidant activity, the following assays were employed: (1) ferric reducing antioxidant power (FRAP), (2) 2,2-diphenyl-1-picrylhydrazyl (DPPH), and (3) 2,2′-azinobis-(3-ethylbenzothiazoline-6-sulfonic acid) (ABTS). In FRAP, measurements were conducted at 593 nm. Radical-scavenging activity was determined via measurements of DPPH at 517 nm. Finally, ABTS was determined with the use of an ABTS solution, with measurements taken at 734 nm. All protocols included the use of Trolox ((±)-6-hydroxy-2,5,7,8-tetramethylchromane-2-carboxylic acid as a positive control. Results for antioxidant activities were expressed as mg Trolox g^−1^ FW, based on a Trolox standard curve [63].

Determination of leaf and fruit tissue hydrogen peroxide (H_2_O_2_) and lipid peroxidation in terms malondialdehyde content (MDA) content was previously described [62] using pooled tissue of each treatment (*n* = 5 for leaves and *n* = 7 for fruits). The H_2_O_2_ concentration was evaluated based on a standard curve ranging from 5 to 1000 μM H_2_O_2_ and measured at 390 nm. Results were expressed as μmol H_2_O_2_ g^−1^ FW. Lipid peroxidation was assessed in terms MDA was determined at 532 nm and corrected at 600 nm. The amount of MDA was determined using the extinction coefficient of 155 mM^−1^ cm^−1^. Results were expressed as nmol MDA g^−1^ FW.

### 4.7. Fruit Quality Assessment and Macroscopic Evaluation

Fruit quality attributes were examined in sever (*n *= 7) replications. Fruit color measurements were conducted as described previously [64] using the Hunter Lab System and a colorimeter (CR400, Konica Minolta Inc., Osaka, Japan). The L*, a*, and b* values of the CIELAB uniform color space were recorded at 4 points of each fruit, where L* represents brightness/lightness (0: black/100: white), a* represents greenness/redness (−a*: greenness and + a*: redness), and b* represents blueness/yellowness (-b*: blueness and + b*: yellowness). Using these measurements, chroma value (*C*), color index (*CI*), whiteness index, and browning index (*BI*) were calculated using the following equations:

(1) $$C={\left(a^{*2}+b^{*2}\right)}^{1/2}$$

(2) $$CI=\frac{a^{*}\times 1000}{L^{*}\times b^{*}}$$

(3) $$WI=1{00-\lfloor {\left(100-L^{*}\right)}^{2}+a^{*2}+b^{*2}\rfloor }^{1/2}$$

(4) $$BI=\frac{100\times (X-0.31)}{0.17}\;\mathrm{where}\;X=\frac{\left(a^{*}+1.75\times L^{*}\right)}{\left(5.645\times L^{*}+a^{*}-3.012\times b^{*}\right)}$$

Fruit firmness assessment was conducted as previously detailed [64]. Measurements were conducted on two points on each tomato’s shoulder by applying an 8 mm plunger, using a texture-meter FT 011 (TR Scientific Instruments, Forli, Italy). The amount of force (in Newtons; N) exerted to break through the fruits’ radial pericarp (i.e., surface) was measured at room temperature (22–24 °C). Seven replications of each treatment were recorded.

The total soluble solids (TSS) of tomato fruit were determined with juice obtained from 2–3 pooled fruits for each replication, measured with a temperature-compensated digital refractometer (model Sper Scientific 300017, Sper Scientific Instruments LLC, Scottsdale, AZ, USA) at 20 °C. Results were expressed in percentages. Titratable acidity (TA) was determined by potentiometric titration (Mettler Toledo International Inc., Greifensee, Switzerland) of 5 mL supernatant diluted to 50 mL with distilled water and the addition of 0.1 N NaOH up to a pH of 8.1. Results were expressed as g of citric acid per 1 L juice (g citric acid L^−1^ juice). Finally, the fruit sweetness/ripening index was calculated using the TSS/TA ratio [65].

Carotenoids (lycopene and *β*-carotene) were determined using blended fruit tissue (1.5 g) for each replication, which was homogenized with 20 mL acetone:hexane 4:6 (*v*/*v*). Following sonication and vortexing, the two phases were separated, and the upper phase was measured at 663, 645, 505, and 453 nm using a spectrophotometer, with the 4:6 ratio of acetone:hexane as a reference. Calculations to determine carotenoids contents were as follows [66], and values were expressed as mg g^−1^ FW:

(5) $$Lycopene\;\left(mg\;100\;{mL}^{-1}\;extract\right)=-0.0458\times A_{663}+0.204\times A_{645}+0.372\times A_{505}-0.0806\times A_{453}\;$$

(6) $$\beta -Carotene\;\left(mg\;100\;{mL}^{-1}\;extract\right)=0.216\times A_{663}-1.22\times A_{645}-0.304\times A_{505}+0.452\times A_{453}\;$$

Ascorbic acid (AA) determination was conducted using the 2,6-Dichloroindophenol titrimetric method previously described [65]. An aliquot of 5 mL of pooled tomato juice for each replication was diluted with 45 mL of oxalic acid (0.1%). The sample was then titrated by the dye solution until the color changed. Values were expressed as mg AA g^−1^ FW.

Fresh produce marketability, aroma, and appearance were recorded on a 1–10 scale; 1: not marketable quality (i.e., malformation, wounds, infection); 3: low marketable with malformation; 5: marketable with few defects i.e., small size, decolorization (medium quality); 8: marketable (good quality); 10: marketable with no defects (extra quality) by three evaluators, with results presented as percentages.

### 4.8. Statistical Methods

Statistical analysis utilized the SPSS version 26 (IBM Corporation, New York, NY, USA) program, and data means (leaves and soil measurements with *n*= 5; fruit quality with *n* = 7; plant growth, no-distractive leaf physiology measurements and fruit weight/yield/macroscopic evaluation in all plants and harvested fruits) were compared (± Standard Error, SE) with one-way ANOVA. Duncan’s multiple range tests were employed for significant data (*p* ≤ 0.05). Analyses were conducted separately for treatments without supplementary fertigation and for treatments with supplementary fertigation.

## 5. Conclusions

Bioproducts (BPs) obtained from the anaerobic digestate of biowaste are emerging as promising soil amendments and biostimulants. The current study utilized a BP derived from food waste and gardening residues in the greenhouse cultivation of tomato (*S. lycopersicum* L.) and compared its performance to that of conventional and commercial organic fertilization. Basal fertilization treatments included conventional fertilization (CF), conventional and organic fertilization (CF + OF), BP at 150 kg ha^−1^ (BP), and conventional fertilization and BP at 150 and 300 kg ha^−1^ (CF + BP, CF + 2BP), without or with supplementary fertigation (SF). The application of BP alone resulted in comparable intermediate growth to CF, while the combined use of CF and BP stimulated growth. In addition, final plant growth-related parameters, biomass production, and fruit yield were unaffected by the inclusion of BP compared to mineral fertilization. Interestingly, even though growth indices were consistently lower with BP alone compared to CF application, differences were not statistically significant. Additionally, using BP alone resulted in a decline in the intermediate leaf macronutrient content relative to CF. This was ameliorated with CF + BP use, as it enhanced the leaf macronutrient content over CF. For CF + 2BP, however, this enhancement was only evidenced under supplementary fertigation, likely due to the increased availability of nutrients. The highest final N and K content was observed with the CF relative to BP use. In contrast, BP use generally increased the final P content of leaves and roots. However, a decline in fruit P was evidenced with BP, apparently due to late P availability and imbalanced nutrition. Regardless of fertigation, the phenolic content and antioxidant profile of leaves was enhanced under BP applications, with fruit exhibiting a similar outcome mostly under no supplementary fertigation. In addition, BP use under no supplementary fertigation resulted in increased MDA and decreased H_2_O_2_ in leaves with the application of BP under no supplementary fertigation, indicating active reactive oxygen species scavenging following oxidative stress. In terms of fruit quality, lycopene and TSS were positively influenced by CF + BP, whereas the introduction of supplementary fertigation subsided significant differences. Combining CF and BP improved tomato fruit quality and without diminishing growth. Although BP alone exhibited suboptimal performance, supplementary fertigation could enhance its efficacy. Ultimately, BPs may be moderately applied (i.e., 150 kg ha^−1^) as supplements to conventional fertilization, contributing to improved production and quality. Interestingly, their application adheres to the regulatory framework of the European Union, with their production being environmentally sustainable. In addition, the use of BPs in commercial agriculture could allow for promoting produce quality at a low cost with minimal impacts to the environment. However, further studies are required to explore the application of BP alone at higher rates as well as the recurring application of BPs in crops, and to directly compare them with common biostimulant products. Additional research may focus on the improvement of BP application rates and its use as a sole fertigation solution as a potential substitute for synthetic fertilizers.

## Figures and Tables

**Table 1:** The effects of CF (conventional), CF + OF (conventional and organic) BP (bioproduct; 150 kg ha^−1^) CF + BP (conventional with bioproduct; 150 kg ha^−1^), CF + 2BP (conventional with bioproduct; 300 kg ha^−1^) basal fertilization, without or with supplementary NPK fertigation (SF) on soil pH, electrical conductivity (EC; mS cm^−1^), CaCO_3_ (%), organic matter content (%), and macronutrient content (N, P, K, Na; g kg^−1^) following cultivation.

	pH	EC	Organic Matter	CaCO_3_	N	P	K	Na
**CF**	7.73 ± 0.03 cd	2.71 ± 0.08 b	1.11 ± 0.07 a	30.98 ± 0.53 a	0.66 ± 0.04 a	0.022 ± 0.004 a	0.18 ± 0.00 a	0.55 ± 0.00 c
**CF + OF**	7.90 ± 0.01 b	2.56 ± 0.07 b	1.04 ± 0.02 ab	28.33 ± 0.41 c	0.66 ± 0.04 a	0.019 ± 0.000 a	0.16 ± 0.00 b	0.55 ± 0.01 c
**BP**	7.97 ± 0.02 a	2.32 ± 0.08 c	1.06 ± 0.02 ab	29.90 ± 0.36 ab	0.69 ± 0.04 a	0.009 ± 0.001 b	0.19 ± 0.00 a	0.48 ± 0.01 d
**CF + BP**	7.71 ± 0.01 d	3.29 ± 0.10 a	0.95 ± 0.02 b	29.14 ± 0.39 bc	0.67 ± 0.02 a	0.018 ± 0.001 a	0.17 ± 0.00 b	0.59 ± 0.01 b
**CF + 2 BP**	7.77 ± 0.01 c	3.13 ± 0.07 a	0.95 ± 0.05 b	28.76 ± 0.39 bc	0.73 ± 0.05 a	0.018 ± 0.002 a	0.19 ± 0.00 a	0.62 ± 0.01 a
**CF/SF**	7.86 ± 0.02 C	2.28 ± 0.03 C	1.05 ± 0.01 A	29.49 ± 0.13 AB	0.72 ± 0.01 AB	0.025 ± 0.001 AB	0.17 ± 0.00 B	0.57 ± 0.01 B
**CF + OF/SF**	7.90 ± 0.01 B	2.44 ± 0.05 B	1.06 ± 0.05 A	29.61 ± 0.46 AB	0.63 ± 0.01 C	0.030 ± 0.001 AB	0.18 ± 0.00 A	0.54 ± 0.01 C
**BP/SF**	7.89 ± 0.00 BC	2.07 ± 0.05 D	1.04 ± 0.04 A	29.90 ± 0.45 AB	0.68 ± 0.01 BC	0.023 ± 0.001 B	0.18 ± 0.00 A	0.55 ± 0.01 C
**CF + BP/SF**	7.73 ± 0.00 D	3.24 ± 0.04 A	0.82 ± 0.07 B	30.24 ± 0.51 A	0.67 ± 0.02 BC	0.033 ± 0.006 A	0.16 ± 0.00 C	0.63 ± 0.00 A
**CF + 2BP/SF**	8.04 ± 0.02 A	1.91 ± 0.07 E	1.01 ± 0.05 A	28.88 ± 0.23 B	0.78 ± 0.04 A	0.033 ± 0.001 A	0.18 ± 0.00 A	0.59 ± 0.01 B

Values represent mean ± standard error (*n *= 5). Values followed by different lowercase letters (without supplementary fertigation) and uppercase letters (with supplementary fertigation-SF) differ significantly (*p* < 0.05). Mean values with the same lettering constitute no significant differences.

**Table 2:** The effects of CF (conventional), CF + OF (conventional and organic) BP (bioproduct; 150 kg ha^−1^) CF + BP (conventional with bioproduct; 150 kg ha^−1^), CF + 2BP (conventional with bioproduct; 300 kg ha^−1^) basal fertilization, without or with supplementary NPK fertigation (SF) on growth-related parameters—plant height (cm), leaf number, base and mid stem thickness (mm), soil plant analysis development (SPAD,) and chlorophyll fluorescence (F_v_/F_m_)—during the intermediate and final stage of the growing period.

**Intermediate Stage**						
	**Plant Height**	**Leaf Number**	**Base Stem Thickness**	**Mid Stem Thickness**	**SPAD**	**F_v_/F_m_**
**CF**	108.63 ± 3.75 b	15.25 ± 0.41 ab	10.76 ± 0.27 a	n.m.	101.76 ± 8.02 a	0.76 ± 0.01 a
**CF + OF**	113.88 ± 1.09 ab	16.25 ± 0.49 ab	10.55 ± 0.41 a	n.m.	108.41 ± 4.20 a	0.77 ± 0.01 a
**BP**	111.50 ± 3.00 ab	15.00 ± 0.50 b	9.52 ± 0.13 b	n.m.	93.08 ± 9.38 a	0.76 ± 0.01 a
**CF + BP**	119.13 ± 1.09 a	16.50 ± 0.19 a	9.45 ± 0.23 b	n.m.	94.41 ± 5.29 a	0.76 ± 0.01 a
**CF + 2 BP**	106.75 ± 4.91 b	15.50 ± 0.63 ab	10.56 ± 0.23 a	n.m.	100.44 ± 4.59 a	0.74 ± 0.01 a
**CF/SF**	117.88 ± 1.79 A	16.25 ± 0.25 A	10.87 ± 0.37 AB	n.m.	110.39 ± 4.53 AB	0.77 ± 0.01 A
**CF + OF/SF**	109.67 ± 2.91 B	15.78 ± 0.32 AB	10.47 ± 0.31 B	n.m.	114.58 ± 3.65 A	0.79 ± 0.01 A
**BP/SF**	114.22 ± 3.20 AB	15.00 ± 0.65 B	10.00 ± 0.31 B	n.m.	96.06 ± 9.25 B	0.75 ± 0.01 A
**CF + BP/SF**	117.56 ± 1.02 A	16.67 ± 0.29 A	10.72 ± 0.33 B	n.m.	99.34 ± 4.77 AB	0.76 ± 0.01 A
**CF + 2BP/SF**	118.22 ± 1.94 A	16.33 ± 0.41 A	11.73 ± 0.24 A	n.m.	92.53 ± 5.38 B	0.76 ± 0.01 A
**Final Stage**						
	**Plant Height**	**Leaf Number**	**Base Stem Thickness**	**Mid Stem Thickness**	**SPAD**	**F_v_/F_m_**
**CF**	195.63 ± 7.78 a	18.88 ± 1.13 a	13.03 ± 0.57 a	6.61 ± 0.48 a	65.36 ± 5.40 a	0.73 ± 0.01 a
**CF + OF**	199.00 ± 15.01 a	18.00 ± 1.89 a	12.29 ± 0.66 a	6.22 ± 0.41 ab	52.69 ± 6.68 a	0.70 ± 0.02 a
**BP**	185.13 ± 6.00 a	15.88 ± 0.72 a	10.48 ± 0.32 b	5.07 ± 0.30 b	58.21 ± 5.89 a	0.71 ± 0.01 a
**CF + BP**	188.88 ± 5.42 a	17.00 ± 0.85 a	11.63 ± 0.40 ab	5.83 ± 0.26 ab	59.96 ± 5.42 a	0.73 ± 0.02 a
**CF + 2BP**	174.13 ± 10.94 a	16.50 ± 0.82 a	12.16 ± 0.41 a	5.85 ± 0.51 ab	65.78 ± 6.31 a	0.71 ± 0.01 a
**CF/SF**	195.13 ± 9.25 A	17.38 ± 1.16 A	12.16 ± 0.16 A	5.86 ± 0.36	67.35 ± 4.85 A	0.75 ± 0.01 A
**CF + OF/SF**	188.33 ± 4.17 A	18.44 ± 0.60 A	11.76 ± 0.34 AB	5.72 ± 0.44	64.53 ± 4.60 A	0.72 ± 0.02 AB
**BP/SF**	186.88 ± 5.80 A	16.75 ± 0.73 A	10.85 ± 0.37 B	5.42 ± 0.67	57.48 ± 2.97 A	0.71 ± 0.01 AB
**CF + BP/SF**	190.11 ± 6.03 A	17.89 ± 0.95 A	12.26 ± 0.46 A	5.73 ± 0.27	60.08 ± 4.08 A	0.70 ± 0.02 B
**CF + 2BP/SF**	200.33 ± 7.32 A	17.44 ± 0.94 A	12.24 ± 0.30 A	6.26 ± 0.70	69.21 ± 6.07 A	0.72 ± 0.01 AB

Values represent mean ± standard error (in all plants/treatment). Values followed by different lowercase letters (without supplementary fertigation) and uppercase letters (with supplementary fertigation-SF) differ significantly (*p* < 0.05). Mean values with the same lettering constitute no significant difference. n.m.: indicates not measured.

**Table 3:** The effects of CF (conventional), CF + OF (conventional and organic) BP (bioproduct; 150 kg ha^−1^) CF + BP (conventional with bioproduct; 150 kg ha^−1^), CF + 2BP (conventional with bioproduct; 300 kg ha^−1^) basal fertilization, without or with supplementary NPK fertigation (SF) on overall fresh weight (FW) of tomato plant leaves (g), and stem (g), fruit (kg) and total biomass (kg) per plant, and dry matter content (DM) of leaves and roots (%).

	Leaves FW	Stem FW	Plant FW	Leaf DM	Root DM
**CF**	542.51 ± 80.95 ab	244.84 ± 34.23 ab	2.36 ± 0.35 a	18.41 ± 0.14 ab	12.87 ± 0.44 ab
**CF + OF**	627.50 ± 130.34 a	277.60 ± 46.76 a	2.74 ± 0.59 a	17.72 ± 0.13 b	12.12 ± 0.27 b
**BP**	346.70 ± 51.49 b	160.89 ± 15.61 b	1.75 ± 0.29 a	18.81 ± 0.16 ab	14.16 ± 0.35 a
**CF + BP**	452.13 ± 7.07 ab	202.97 ± 6.35 ab	1.85 ± 0.07 a	19.10 ± 0.46 ab	11.83 ± 0.47 b
**CF + 2BP**	409.87 ± 45.45 ab	194.87 ± 22.42 ab	1.78 ± 0.14 a	20.29 ± 1.00 a	12.55 ± 0.61 b
**CF/SF**	548.77 ± 50.04 AB	240.13 ± 19.10 AB	2.27 ± 0.21 AB	19.12 ± 0.42 A	13.17 ± 0.31 A
**CF + OF/SF**	481.74 ± 34.70 AB	207.03 ± 12.31 B	1.93 ± 0.16 AB	18.11 ± 0.25 B	14.36 ± 0.97 A
**BP/SF**	437.74 ± 48.51 B	205.95 ± 21.36 B	1.77 ± 0.07 B	19.09 ± 0.00 A	14.85 ± 0.69 A
**CF + BP/SF**	517.06 ± 34.77 AB	226.59 ± 13.43 AB	1.98 ± 0.11 AB	18.53 ± 0.19 AB	13.55 ± 0.77 A
**CF + 2BP/SF**	595.41 ± 59.27 A	267.58 ± 25.07 A	2.52 ± 0.31 A	18.49 ± 0.19 AB	15.32 ± 0.72 A

Values represent mean ± standard error (*n *= at least 7). Values followed by different lowercase letters (without supplementary fertigation) and uppercase letters (with supplementary fertigation-SF) differ significantly (*p* < 0.05). Mean values with the same lettering constitute no significant differences.

**Table 4:** The effects of CF (conventional), CF + OF (conventional and organic), BP (bioproduct; 150 kg ha^−1^), CF + BP (conventional with bioproduct; 150 kg ha^−1^), CF + 2BP (conventional with bioproduct; 300 kg ha^−1^), basal fertilization, without or with supplementary NPK fertigation (SF) on number of marketable fruits, mean fruit fresh weight (FW; g), total yield (kg), and total marketable yield (kg) per plant.

	Fruit No.	Mean Fruit FW	Total Yield FW	Marketable Yield
**CF**	11.57 ± 1.45 a	92.29 ± 9.10 a	1.58 ± 0.24 a	1.09 ± 0.21 a
**CF + OF**	12.14 ± 1.72 a	101.42 ± 12.24 a	1.83 ± 0.41 a	1.35 ± 0.36 a
**BP**	10.00 ± 1.53 a	89.61 ± 7.27 a	1.24 ± 0.23 a	0.91 ± 0.18 a
**CF + BP**	9.14 ± 1.61 a	78.03 ± 6.11 a	1.20 ± 0.08 a	0.72 ± 0.14 a
**CF + 2BP**	8.43 ± 1.25 a	86.03 ± 5.18 a	1.18 ± 0.12 a	0.74 ± 0.14 a
**CF/SF**	11.43 ± 1.13 AB	84.21 ± 7.07 A	1.47 ± 0.15 AB	0.96 ± 0.13 A
**CF + OF/SF**	8.50 ± 1.10 B	88.75 ± 8.52 A	1.24 ± 0.16 AB	0.78 ± 0.14 A
**BP/SF**	8.00 ± 0.85 B	77.48 ± 5.07 A	1.13 ± 0.06 B	0.64 ± 0.11 A
**CF + BP/SF**	9.57 ± 0.87 AB	81.42 ± 6.78 A	1.27 ± 0.09 AB	0.79 ± 0.10 A
**CF + 2BP/SF**	12.25 ± 1.54 A	86.70 ± 7.99 A	1.65 ± 0.24 A	1.09 ± 0.21 A

Values represent mean ± standard error (in all harvested fruits). Values followed by different lowercase letters (without supplementary fertigation) and uppercase letters (with supplementary fertigation-SF) differ significantly (*p* < 0.05). Mean values with the same lettering constitute no significant differences.

**Table 5:** The effects of CF (conventional), CF + OF (conventional and organic) BP (bioproduct; 150 kg ha^−1^) CF + BP (conventional with bioproduct; 150 kg ha^−1^), CF + 2BP (conventional with bioproduct; 300 kg ha^−1^) basal fertilization, without or with supplementary NPK fertigation (SF) on fruit firmness (N), total soluble solids (TSS; %), titratable acidity (TA; % citric acid), sweetness/ripening index (TSS/TA), lycopene (mg 100 g^−1^), *β*-carotene (mg 100 g^−1^), ascorbic acid (mg 100 g^−1^), and macroscopic evaluation (marketability, aroma, appearance) at a scale of 1–10.

	Firmness	TSS	TA	TSS/TA	Lycopene	*β*-Carotene	Ascorbic Acid	Marketability	Aroma	Appearance
**CF**	0.59 ± 0.07 a	5.11 ± 0.32 bc	4.62 ± 0.58 a	1.17 ± 0.10 a	2.46 ± 0.19 b	1.06 ± 0.03 bc	20.02 ± 0.79 a	6.30 ± 0.23 a	7.15 ± 0.10 a	7.07 ± 0.11 ab
**CF + OF**	0.68 ± 0.07 a	4.73 ± 0.18 bc	4.77 ± 0.59 a	1.05 ± 0.09 a	2.74 ± 0.12 ab	1.11 ± 0.05 abc	16.04 ± 0.75 b	5.93 ± 0.41 a	6.93 ± 0.22 ab	7.41 ± 0.21 a
**BP**	0.57 ± 0.09 a	4.51 ± 0.14 c	4.50 ± 0.79 a	1.11 ± 0.11 a	2.26 ± 0.17 b	1.03 ± 0.03 c	17.76 ± 0.49 ab	6.33 ± 0.41 a	6.33 ± 0.14 c	6.78 ± 0.30 b
**CF + BP**	0.76 ± 0.13 a	5.89 ± 0.11 a	5.19 ± 0.66 a	1.26 ± 0.17 a	3.13 ± 0.22 a	1.18 ± 0.03 ab	16.35 ± 1.33 b	6.11 ± 0.26 a	6.67 ± 0.17 bc	6.85 ± 0.17 ab
**CF + 2BP**	0.63 ± 0.09 a	5.30 ± 0.19 b	5.36 ± 0.75 a	1.11 ± 0.15 a	2.60 ± 0.14 b	1.24 ± 0.07 a	19.27 ± 0.63 a	5.41 ± 0.24 a	6.52 ± 0.11 bc	6.78 ± 0.14 b
**CF/SF**	0.73 ± 0.10 AB	5.71 ± 0.22 A	5.81 ± 0.62 A	1.05 ± 0.12 A	2.80 ± 0.18 A	1.19 ± 0.03 A	20.42 ± 0.77 B	5.59 ± 0.31 A	6.74 ± 0.25 A	6.63 ± 0.18 A
**CF + OF/SF**	0.52 ± 0.05 B	4.84 ± 0.10 B	4.76 ± 0.62 A	1.10 ± 0.11 A	2.32 ± 0.08 A	1.20 ± 0.04 A	21.35 ± 0.34 B	6.41 ± 0.43 A	6.22 ± 0.12 B	6.82 ± 0.17 A
**BP/SF**	0.64 ± 0.09 AB	4.90 ± 0.30 B	4.75 ± 0.52 A	1.07 ± 0.07 A	2.45 ± 0.24 A	1.16 ± 0.03 A	20.50 ± 0.28 B	6.52 ± 0.34 A	6.48 ± 0.13 AB	6.96 ± 0.15 A
**CF + BP/SF**	0.89 ± 0.13 A	5.91 ± 0.19 A	5.14 ± 0.41 A	1.20 ± 0.11 A	2.83 ± 0.27 A	1.10 ± 0.03 A	23.88 ± 0.57 A	6.33 ± 0.34 A	6.63 ± 0.07 AB	7.15 ± 0.16 A
**CF + 2BP/SF**	0.54 ± 0.08 B	5.43 ± 0.25 AB	4.40 ± 0.62 A	1.34 ± 0.15 A	2.62 ± 0.15 A	1.15 ± 0.03 A	23.09 ± 0.78 A	5.96 ± 0.24 A	6.67 ± 0.10 AB	6.93 ± 0.22 A

Values represent mean ± standard error (*n *= 7). Values followed by different lowercase letters (without supplementary fertigation) and uppercase letters (with supplementary fertigation-SF) differ significantly (*p* < 0.05). Mean values with the same lettering constitute no significant differences.

## Data Availability

The authors declare data availability only upon request.

## Supplementary Materials

The following supporting information can be downloaded at: https://www.mdpi.com/article/10.3390/plants14203212/s1, Figure S1: Temperature (°C) and relative humidity (%) deviation during the experimental period, Table S1: Properties of the soil used in the experiment, Table S2: Characteristics of the bioproduct (BP) used in the current experiment, Table S3: The treatments examined in the current experiment, Table S4: The fertilizer applications of the current experiment, Table S5: The effects of fertilizer application on leachates’ pH, electrical conductivity (EC; mS cm^−1^), and macronutrient concentrations (P, K, Na; g L^−1^) following the cultivation period, Table S6: The effects of CF (conventional), CF + OF (conventional and organic) BP (bioproduct; 150 kg ha^−1^) CF + BP (conventional with bioproduct; 150 kg ha^−1^), CF + 2BP (conventional with bioproduct; 300 kg ha^−1^) basal fertilization, without or with supplementary NPK fertigation (SF) on leaf chlorophyll a (Chl a), chlorophyll b (Chl b), total chlorophylls (Total Chl) and total carotenoids (Total Car), Table S7: The effects of CF (conventional), CF + OF (conventional and organic) BP (bioproduct; 150 kg ha^−1^) CF + BP (conventional with bioproduct; 150 kg ha^−1^), CF + 2BP (conventional with bioproduct; 300 kg ha^−1^) basal fertilization, without or with supplementary NPK fertigation (SF) on fruit color; *L** value, *a** value, *b** value, hue (h) (o), chroma value (C) color index (CI), whiteness index (WI), and browning index (BI), Table S8: Tomato fruit symptoms (Blossom end rot-BER, cracking, russeting, wounding) recorded during the experiment.
