# Supplementary material for: Application of Municipal Biowaste-Derived Products in Tomato Cultivation for Enhanced Fruit Quality Attributes and Nutritional Profile
Source: Plants (Basel). 2025 Oct 19;14(20):3212. doi: 10.3390/plants14203212 (PMC12567243; doi:10.3390/plants14203212)
Supplement: Supplementary file 1 [file plants-14-03212-s001.zip › plants-3897355-supplementary.pdf]

Supplementary material

Table S1. Properties of the soil used in the experiment.

Attribute	Value	Attribute	Value
**pH**	7.29±0.35	**N (g kg^{-1})**	0.54±0.86
**EC (mS cm^{-1})**	2.09±0.12	**P (g kg^{-1})**	0.01±0.00
**$CaCO_3$ (%)**	28.55±0.35	**K (g kg^{-1})**	0.20±0.01
**Organic matter (%)**	0.53±0.04	**Ca (g kg^{-1})**	6.59±0.15
		**Mg (g kg^{-1})**	1.14±0.03
		**Na (g kg^{-1})**	0.33±0.00

Values represent mean ± standard error.

Table S2. Characteristics of the bioproduct (BP) used in the current experiment.

Attribute	Value	Attribute	Value
pH	9.06±0.04	N (g kg^{-1})	42.17±0.29
Electrical conductivity (mS cm^{-1})	6.67±0.01	P (g kg^{-1})	11.98±0.06
Humidity (%)	27.31±0.30	K (g kg^{-1})	35.97±1.54
Organic matter content (%)	68.09±0.03	Ca (g kg^{-1})	31.33±1.45
Organic C (%)	39.50±0.02	Mg (g kg^{-1})	6.45±0.29
		Na (g kg^{-1})	1.83±0.05

Values represent mean ± standard error.

Table S3. The treatments examined in the current experiment

Code	Treatment
**CF**	Conventional fertilization (N:P:K)
**CF/SF**	Conventional fertilization (N:P:K) + supplementary fertigation
**CF+OF**	Conventional fertilization (N:P:K) & Organic fertilizer (Phenix 6-8-15, Hello Nature International Srl, Italy)
**CF+OF/S**	Conventional fertilization (N:P:K) & Organic fertilizer (Phenix 6-8-15, Hello Nature International Srl, Italy) + supplementary fertigation
**BP**	BP (150kg ha^{-1})
**BP/SF**	BP (150 kg ha^{-1}) + supplementary fertigation
**CF+BP**	Conventional fertilization (N:P:K) & BP (150kg ha^{-1})
**CF+BP/SF**	Conventional fertilization (N:P:K) & BP (150kg ha^{-1}) + supplementary fertigation
**CF+2BP**	Conventional fertilization (N:P:K) & BP (300kg ha^{-1})
**CF+2BP/SF**	Conventional fertilization (N:P:K) & BP (300kg ha^{-1}) + supplementary fertigation

Table S4. The fertilizer applications of the current experiment.

Code	NH_4NO_3 (g kg^{-1})	Triple superphosphate (g kg^{-1})	Potassium sulphate (g kg^{-1})	LIFE BP (g kg^{-1})	Phenix (g kg^{-1})
**CF**	0.4	0.1536	0.2857	-	-
**CF+OF**	0.4	0.1536	0.2857	-	0.3571
**BP**	-	-	-	1.5	-
**CF+BP**	0.4	0.1536	0.2857	1.5	-
**CF+2BP**	0.4	0.1536	0.2857	3	-

Table S5. The effects of fertilizer application (CF; conventional fertilization, CF+OF; conventional and organic fertilization, BP; BP at 150 kg ha^{-1}, CF+BP; conventional fertilization and BP at 150 kg ha^{-1}, CF+2BP; conventional fertilization and BP at 300 kg ha^{-1}), without

 https://doi.org/10.3390/xxxxx

or with supplementary NPK fertigation (SF) on leachates' pH, electrical conductivity (EC; mS cm^{-1}), and macronutrient concentrations (P, K, Na; g L^{-1}) following the cultivation period.

	**pH**	**EC**	**P**	**K**	**Na**
**CF**	7.21±0.15b	1.91±0.04a	0.004±0.001a	0.006±0.002a	0.62±0.34a
**CF+OF**	7.63±0.03a	2.69±0.31a	0.005±0.001a	0.006±0.001a	0.39±0.03a
**BP**	7.54±0.07a	3.65±1.49a	0.004±0.001a	0.007±0.002a	0.50±0.19a
**CF+BP**	7.61±0.07a	5.18±0.72a	0.004±0.001a	0.006±0.002a	0.67±0.08a
**CF+2BP**	7.61±0.06a	4.16±1.94a	0.005±0.001a	0.007±0.002a	0.53±0.20a
**CF/SF**	7.41±0.08B	4.07±0.56A	0.004±0.000A	0.005±0.002A	0.52±0.01A
**CF+OF/SF**	7.71±0.04A	2.66±0.35A	0.004±0.001A	0.008±0.004A	0.43±0.03A
**BP/SF**	7.60±0.04A	4.42±0.69A	0.005±0.002A	0.008±0.001A	0.53±0.09A
**CF+BP/SF**	7.53±0.06AB	5.69±2.94A	0.005±0.001A	0.005±0.001A	0.61±0.23A
**CF+2BP/SF**	7.68±0.01A	3.30±0.31A	0.003±0.001A	0.007±0.001A	0.50±0.08A

Values represent mean ± standard error (n = 4). Values followed by different lowercase letters (with supplementary fertigation) and uppercase letters (without supplementary fertigation) differ significantly (P < 0.05). Mean values with the same lettering constitute no significant differences.

Table S6. The effects of CF (conventional), CF+OF (conventional and organic) BP (bioproduct; 150 kg ha^{-1}) CF+BP (conventional with bioproduct; 150 kg ha^{-1}), CF+2BP (conventional with bioproduct; 300 kg ha^{-1}) basal fertilization, without or with supplementary NPK fertigation (SF) on leaf chlorophyll a (Chl a), chlorophyll b (Chl b), total chlorophylls (Total Chl) and total carotenoids (Total Car).

	**Chl a**	**Chl b**	**Total Chl**	**Total Car**
**CF**	0.85±0.07a	0.34±0.10a	1.18±0.12a	0.09±0.01a
**CF+OF**	0.65±0.07abc	0.31±0.03a	0.95±0.10a	0.09±0.01a
**BP**	0.77±0.04ab	0.40±0.07a	1.17±0.10a	0.09±0.01a
**CF+BP**	0.58±0.09bc	0.29±0.05a	0.87±0.14a	0.08±0.01a
**CF+2BP**	0.53±0.08c	0.30±0.03a	0.83±0.11a	0.11±0.02a
**CF/SF**	0.68±0.03A	0.33±0.02A	1.01±0.04A	0.09±0.00A
**CF+OF/SF**	0.71±0.07A	0.34±0.04A	1.05±0.10A	0.09±0.00A
**BP/SF**	0.63±0.10A	0.32±0.06A	0.94±0.16A	0.09±0.02A
**CF+BP/SF**	0.63±0.05A	0.31±0.03A	0.93±0.07A	0.09±0.01A
**CF+2BP/SF**	0.57±0.03A	0.27±0.02A	0.84±0.05A	0.08±0.01A

Values represent mean ± standard error (n = 4). Values followed by different lowercase letters (with supplementary fertigation) and uppercase letters (without supplementary fertigation) differ significantly (P < 0.05). Mean values with the same lettering constitute no significant differences.

Table S7. The effects of CF (conventional), CF+OF (conventional and organic) BP (bioproduct; 150 kg ha^{-1}) CF+BP (conventional with bioproduct; 150 kg ha^{-1}), CF+2BP (conventional with bioproduct; 300 kg ha^{-1}) basal fertilization, without or with supplementary NPK fertigation (SF) on fruit color; L^* value, a^* value, b^* value, hue (h) (o), chroma value (C) color index (CI), whiteness index (WI), and browning index (BI).

	**Color L^***	**Color a^***	**Color b^***	**Hue (h) (o)**	**Chroma value (C)**	**Color index (CI)**	**Whiteness index (WI)**	**Browning index (BI)**
**CF**	38.90±0.31a	27.50±0.47a	24.73±0.40a	41.98±0.60a	37.00±0.49b	28.66±0.74a	28.56±0.23a	143.36±1.67a
**CF+OF**	39.19±0.56a	27.73±0.69a	24.99±0.73a	42.00±0.75a	37.36±0.87ab	28.52±1.03a	28.57±0.21a	143.72±2.35a
**BP**	38.92±0.52a	28.01±0.98a	25.01±0.88a	41.76±0.71a	37.57±1.23ab	28.93±0.95a	28.20±0.40a	145.50±4.09a
**CF+BP**	40.43±0.55a	29.80±1.33a	26.83±0.81a	42.16±1.43a	40.20±1.19a	27.77±1.69a	28.07±0.68a	151.31±3.52a
**CF+2BP**	39.77±0.44a	29.21±0.66a	26.11±0.79a	41.75±0.77a	39.21±0.88ab	28.34±0.98a	28.08±0.28a	149.61±3.13a
**CF/SF**	40.33±0.56A	27.97±0.75B	27.17±0.77A	44.16±1.23A	39.06±0.69AE	25.87±1.43A	28.65±0.42A	151.13±2.19A
**CF+OF/SF**	39.83±0.53A	27.18±1.01B	26.18±0.71A	43.99±1.50A	37.84±0.72B	26.42±1.53A	28.89±0.53A	146.71±2.24A
**BP/SF**	40.19±0.68A	29.01±1.02AE	27.27±1.05A	43.22±1.37A	39.90±1.12AE	26.91±1.59A	28.02±0.43A	153.79±3.45A

**CF+BP/SF**	40.51±0.38A	30.85±0.69A	26.63±0.53A	40.83±0.58A	40.77±0.77A	28.65±0.67A	27.84±0.19A	151.31±1.86A
**CF+2BP/SF**	40.75±0.56A	29.23±0.58AB	27.31±0.93A	42.97±0.98A	40.05±0.85AB	26.60±1.22A	28.43±0.27A	151.69±2.99A

Values represent mean ± standard error (*n* = 4). Values followed by different lowercase letters (with supplementary fertigation) and uppercase letters (without supplementary fertigation) differ significantly ($P < 0.05$). Mean values with the same lettering constitute no significant differences.

Table S8. Tomato fruit symptoms (Blossom end rot-BER, cracking, russeting, wounding) recorded during the experiment.

	BER	Cracking	Russeting	Wounding
**CF**	18.93±7.53a	7.35±5.18a	2.98±1.97a	0.00±0.00a
**CF+OF**	10.33±3.51a	14.26±4.19a	2.18±1.50a	0.00±0.00a
**BP**	23.84±12.60a	2.86±2.86a	6.21±2.96a	1.02±1.02a
**CF+BP**	17.13±5.79a	4.47±2.18a	0.00±0.00a	0.00±0.00a
**CF+2BP**	13.23±5.74a	8.23±3.12a	6.21±2.96a	1.43±1.43a
**CF/SF**	25.60±6.93A	7.05±2.64A	1.79±1.79A	0.00±0.00A
**CF+OF/SF**	24.77±4.80A	9.78±3.49A	2.50±2.50A	0.00±0.00A
**BP/SF**	17.76±6.72A	7.04±3.83A	2.08±2.08A	0.00±0.00A
**CF+BP/SF**	15.80±5.79A	10.32±5.87A	2.73±1.76A	0.00±0.00A
**CF+2BP/SF**	23.43±4.73A	8.47±3.46A	2.76±1.38A	0.00±0.00A

Values represent mean ± standard error (all fruits). Mean values with the same lettering constitute no significant differences.

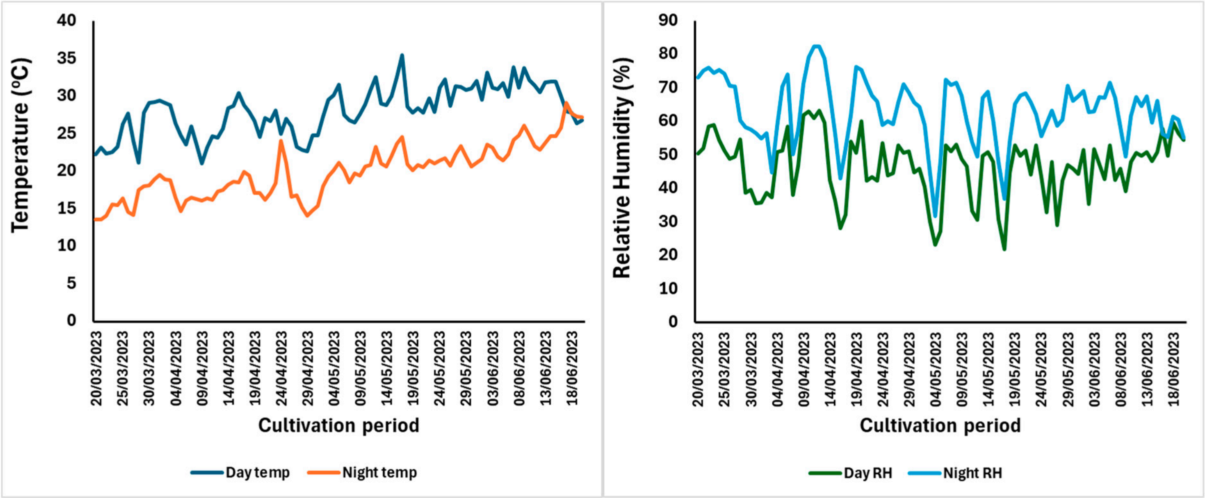

Figure S1. Temperature (°C) and relative humidity (%) deviation during the experimental period.
